# Acceptability and Potential Impact of the #chatsafe Suicide Postvention Response Among Young People Who Have Been Exposed to Suicide: Pilot Study

**DOI:** 10.2196/44535

**Published:** 2023-05-19

**Authors:** Louise La Sala, Jane Pirkis, Charlie Cooper, Nicole T M Hill, Michelle Lamblin, Gowri Rajaram, Simon Rice, Zoe Teh, Pinar Thorn, Rifat Zahan, Jo Robinson

**Affiliations:** 1 Orygen Centre for Youth Mental Health The University of Melbourne Parkville Australia; 2 Centre for Mental Health The University of Melbourne Parkville Australia; 3 Telethon Kids Institute Perth Australia; 4 Department of Computer Science University of Saskatchewan Saskatoon, SK Canada

**Keywords:** youth, suicide, social media, suicide postvention, suicide prevention, contagion, postvention

## Abstract

**Background:**

Young people are more likely to be affected by suicide contagion, and there are concerns about the role social media plays in the development and maintenance of suicide clusters or in facilitating imitative suicidal behavior. However, social media also presents an opportunity to provide real-time and age-appropriate suicide prevention information, which could be an important component of suicide postvention activities.

**Objective:**

This study aimed to test an intervention designed to equip young people to communicate safely online about suicide (#chatsafe) with a sample of young people who had recently been exposed to a suicide or suicide attempt, with a view to determining the role social media can play as part of a postvention response.

**Methods:**

A sample of 266 young people from Australia, aged 16 to 25 years, were recruited to participate in the study. They were eligible if they had been exposed to a suicide or knew of a suicide attempt in the past 2 years. All participants received the #chatsafe intervention, which comprised 6 pieces of social media content that were sent to them weekly via direct message through Instagram, Facebook, or Snapchat. Participants were assessed on a range of outcome measures (social media use, willingness to intervene against suicide, internet self-efficacy, confidence, and safety when communicating about suicide on social media platforms) at baseline, immediately after the intervention, and at 4-week follow-up.

**Results:**

After the 6-week #chatsafe intervention, participants reported substantial improvements in their willingness to intervene against suicide online, their internet self-efficacy, and their perceived confidence and safety when communicating about suicide online. Overall, the participants reported that it was appropriate to receive the #chatsafe intervention via social media, and no iatrogenic effects were recorded.

**Conclusions:**

The findings suggest that it is safe and acceptable to disseminate suicide prevention information entirely via social media among young people who have recently been exposed to a suicide or suicide attempt. Interventions such as #chatsafe could potentially mitigate the risk of distress and future suicidal behavior in young people by improving the quality and safety of online communication about suicide and, as such, can be an important component of delivering a postvention response to young people.

## Introduction

### Background

Suicide is the leading cause of death among young people in Australia [1] and the second leading cause worldwide [2]. Although overall suicide rates have been decreasing in recent decades [3], this is not the case for young people for whom suicide rates have steadily increased in many parts of the world [4].

Youth suicides are between 2 and 4 times more likely to form part of a suicide cluster than adult suicides, with approximately 2.5% of youth suicides in Australia estimated to be part of a suicide cluster [5,6]. Suicide clusters are defined as a group of suicides that occur closer together in time and space than would normally be expected based on either statistical prediction or community expectation [7]. While the underlying mechanisms that facilitate the development and maintenance of suicide clusters are not well understood, one of the most common suggestions is that contagion or imitation occurs via social learning, where the suicide of one person may lead others who relate or identify with that person to engage in similar behavior [8,9]. Those thought to be most susceptible to this process are adolescents and young people [10] as well as those who are geographically close to the person who has died by suicide (eg, witness the death), those who identify most closely with them, and those who are already susceptible in some way, (eg, have a history of suicidality) [11].

One group who may be particularly susceptible to contagion are those who have been bereaved by, or exposed to, a suicide [12]. In a nationwide study conducted in Australia, almost 7% of young people aged 10 to 24 years who died by suicide had been exposed to the suicide of a friend or family member at some point in their lifetime [13], and exposure to a suicide has been shown to increase subsequent risk by approximately 300% [14]. Just as exposure can occur in person through connected networks, it can also occur via media (both traditional media and online media). Certain types of media reporting of suicide have been shown to increase imitative suicidal behavior in others [15], and being exposed to suicide in a way that glamourizes suicidal behavior or garners a lot of attention (eg, public outpourings of how much someone will be missed) is thought to play a role in this [15].

Concerns relating to the impact of exposure to suicide have been heightened in the age of social media [9,16]. This is unsurprising, given the amount of time young people typically spend online and the speed at which unregulated and potentially distressing information about suicide can spread [17,18]. Concurrent with research findings for traditional media, exposure to graphic or distressing information about suicide on social media has been linked to an increase in suicidal thoughts and behaviors among young people [19]. This is worrying, given the rates at which young people are exposed to suicide-related content online, including graphic descriptions of suicide and statements encouraging someone to take their own life [20]. While some young people may actively seek suicide-related content online, in many cases, they are inadvertently exposed to this content [19,21,22].

Although exposure to suicide-related content online can be distressing, social media is also an important source of connection and support for young people, including when it comes to communicating about their own experiences with suicide [23,24] and grieving for someone who has died by suicide [25]. Therefore, social media is an important avenue to consider when supporting young people with their own suicidal thoughts and feelings as well as following bereavement by suicide. Indeed, social media platforms provide an opportunity to reach young people with suicide prevention information [20,26,27]; targeted information could be shared with those who have been bereaved by, or exposed to, suicide in an effort to provide support and minimize the spread of harmful or distressing information.

Very little is known about what constitutes the most effective postvention response for young people [28], and even less is known about how best to incorporate social media into those activities [9,29]. Although guidelines exist for implementing a multifaceted postvention response after a suicide has occurred [30-33], no postvention or cluster response strategy currently includes clear guidance for the use of social media. It has been argued that interventions that prevent the spread of harmful suicide-related content, particularly within 90 days of a suicide occurring, may have the potential to reduce the risk of subsequent suicide deaths within that community and provide necessary support to those exposed to the suicide [34]. Given its acceptability and its capacity to reach large numbers of young people quickly, social media could represent an important part of a postvention response.

One intervention that could form part of this response is #chatsafe. #chatsafe comprises a set of evidence-informed guidelines and accompanying social media campaign designed to educate young people about how to communicate safely online about suicide [26,27]. To date, the social media campaign has been viewed by more than 4 million young people worldwide [35]. It was evaluated among a general population sample of young people aged 16 to 25 years in Australia and was shown to increase participants’ perceived internet self-efficacy, confidence, and safety when communicating on social media about suicide. It also increased their willingness to intervene against suicide online [20]. However, to date, it has not been tested among young people who have previously been exposed to a suicide.

### Aims and Hypotheses

The aim of this study was to test the #chatsafe intervention with a sample of young people who had been exposed to a suicide or suicide attempt in the past 2 years.

We hypothesized that, after receiving the #chatsafe intervention, young people who had been exposed to a suicide or suicide attempt in the past 2 years would report an increase in their willingness to intervene against suicide online (hypothesis 1). We also hypothesized that increases would be observed in participants’ perceived internet self-efficacy (hypothesis 2) as well as a greater adherence to communication behavior recommended by the #chatsafe guidelines (hypothesis 3). A further exploratory aim of this study was to investigate the safety and acceptability of the intervention and to determine whether age, gender, or rate of social media use influenced the impact of the #chatsafe intervention.

## Methods

### Design and Setting

This study largely used the same design as the original #chatsafe study [20], except that it sought to specifically recruit young people who had been exposed to a suicide or a suicide attempt (as opposed to the general population of young people).

**Figure 1:**
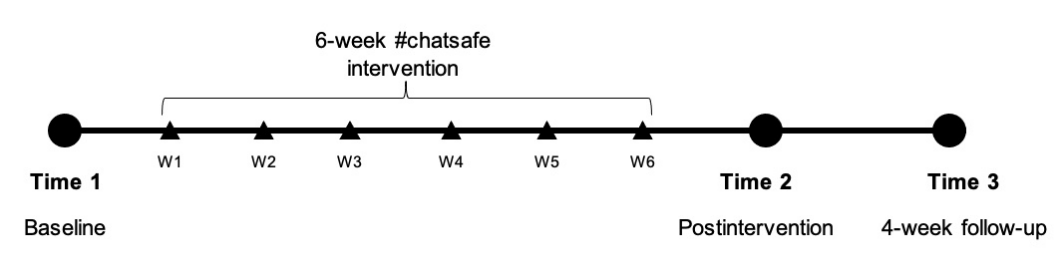
Timeline of the study and #chatsafe intervention. W: week.

It used a prepost study design with a 6-week intervention period. The study was conducted online, and young people were assessed on the primary and secondary outcome variables at 3 time points: baseline (time 1; T1), immediately after the intervention (time 2; T2), and at the 4-week follow-up (time 3; T3). The participants also completed a short weekly survey, from week 1 to week 6. The study timeline is shown in Figure 1.

This study was conducted in Australia between July 2020 and March 2021. It has been reported in accordance with the Template for Intervention Description and Replication (TIDieR) checklist [36].

### Participants

Young people were recruited to the study via targeted advertising on Instagram, Snapchat, and Facebook during the 5-month period from July to November 2020. Young people were eligible to participate if they (1) were aged between 16 and 25 years, inclusive; (2) lived in Australia; (3) had not participated in the previous #chatsafe study; (4) knew of someone who had died by suicide or attempted suicide in the past 2 years (including a friend, family member, or someone in their online or offline communities); and (5) were willing to provide the details of an active Instagram, Snapchat, or Facebook account to the research team to receive the intervention.

After providing consent, all communication with participants took place via direct message through their nominated social media platform. Young people were reimbursed Aus $30 (US $20.13) per completed survey via direct bank transfer.

### Intervention

As described previously, the #chatsafe intervention comprises a set of evidence-informed guidelines that are distributed to young people via a co-designed suite of social media content [20,26,27]. For this study, 2 co-designed workshops were conducted in 2020 to create specific content for young people who had been impacted by a suicide or suicide attempt.

**Figure 2:**
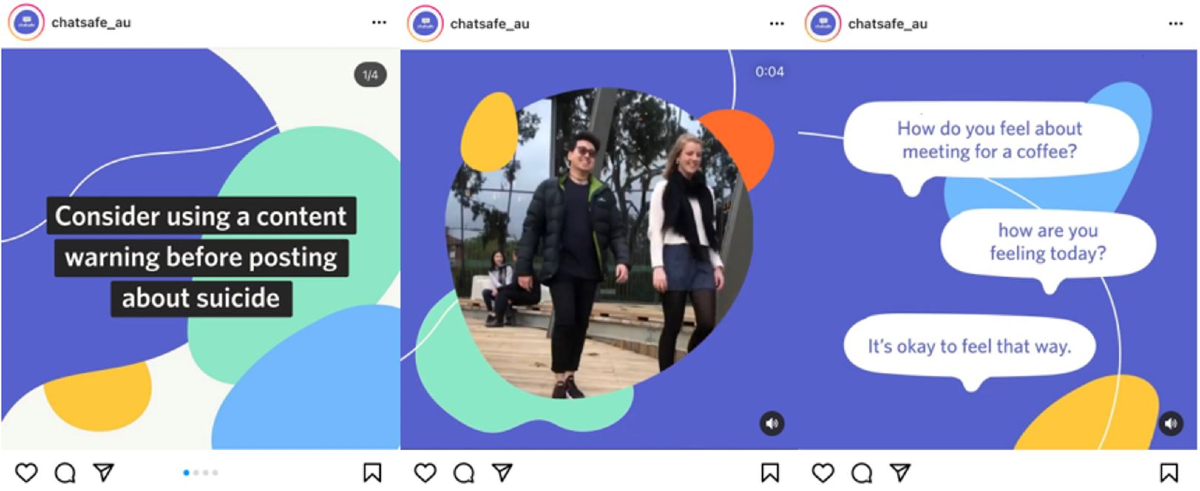
Examples of social media content shared on the #chatsafe social media pages during this study. Left: a text tile encouraging users to consider using a content warning. Middle: a still image of a short video (with no audio) depicting 2 young people “taking a break.” Right: a still image of an animation video discussing how to support someone affected by suicide.

The intervention consisted of a 6-week social media campaign that was shared on the #chatsafe Instagram page [37]. Each week, 3 posts were shared on Instagram, resulting in 18 pieces of content in total. Not only were participants able to view the entire campaign on the public Instagram page but they were also sent 1 post per week via direct message to their preferred social media platform: Instagram, Facebook, or Snapchat. Information about available national support services and a link to a weekly acceptability questionnaire were also sent to participants each week. The intervention is described in Table 1, and specific examples of the content are shown in Figure 2.

**Table 1:** Content theme, content type, and information contained within the content and content copy of the intervention material.

Week	Content theme	Content type	Information contained in content and content copy
1	General introduction to the #chatsafe campaign	Text only	Introducing participants to the #chatsafe guidelines and how the content was developed
2	Safely sharing information about suicide: using trigger and content warnings	Text with digital illustration	Highlighting the importance of using trigger warnings with examples of how to do so
3	Self-care: take a break from social media	Digital illustration	Encouraging participants to take a break from social media after being exposed to upsetting content online
4	Language matters: how to safely talk about suicide online	Text only	Describing the importance of safe language when talking about suicide with examples of how to do so
5	Self-care: take a break from social media	Boomerang (no audio)	Encouraging participants to take a break from social media after being exposed to upsetting content online
6	How to check in on a friend affected by suicide	Animation	Normalizing the difficulty of talking about suicide and providing examples of how to check in on someone who has been affected by suicide

### Study Outcomes and Measures

The primary outcome of interest was participants’ willingness to intervene against suicide at T2, with the 2 subscales from this measure being perceived behavioral control and intent to intervene against suicide [38]. Secondary outcomes included internet self-efficacy [39] and perceived confidence and safety when communicating online about suicide [40] at T2. The measures used to assess these outcomes have been used previously and are described in the study by La Sala et al [20]. In brief, internet self-efficacy comprises 5 domains: reactive and generative (problem-solving and contributing unique information online), organization (organizing information on social media platforms), differentiation (willingness to follow hyperlinks in goal-oriented tasks), search (using advanced search engines), and communication (navigating social networking sites). Adherence to communication behaviors recommended in the #chatsafe guidelines was measured using items from the perceived safety questionnaire (eg, how often they liked, shared, or created a post, including suicide-related information, and how they responded to suicide-related content online) as well as other items recommended in the #chatsafe guidelines (eg, monitoring social media posts and reporting unsafe content) [26].

All data were collected through online self-report surveys at 3 time points using Qualtrics (Figure 1).

At T1, participants also completed a demographic questionnaire assessing age, primary language spoken at home, Aboriginal or Torres Strait Islander identity, gender identity, sexual orientation, student or employment status, and social media use [41].

Acceptability and safety of the #chatsafe intervention were also examined. Acceptability was assessed in 2 ways. First, participants were asked each week to complete a 5-point Likert emoji scale rating their satisfaction with the content sent to them that week [20]. Second, 5 purpose-designed questions assessing the overall acceptability of the 6-week intervention were included in the T2 survey. Safety was measured by the number and nature of serious adverse events and reactions to the content shared by the study team throughout the #chatsafe intervention.

### Data Analysis

To test the primary hypothesis that there would be an increase in scores on both subscales of the willingness to intervene against suicide measure between T1 to T2, regression analyses were used to determine the extent to which the predictor variables (gender, age group, and social media use) could predict the primary outcome relative to no change. The changes in scores from T1 to T2 were grouped based on the magnitude of change from the baseline score, calculated from the SD of the baseline score multiplied by 0.3 (small to medium effect size as per Cohen classification [42]) to derive thresholds for substantial deterioration, no change, and substantial improvement (Multimedia Appendix 1). This standardized difference approach to effect size classification has been used in previous studies [43,44] and was also used to assess changes from T1 to T2 for the Internet Self-Efficacy Scale domains as well as changes from T1 to T3 for both the Willingness to Intervene Against Suicide and Internet Self-Efficacy measures. The thresholds used for these measurements are listed in Table S1 in Multimedia Appendix 1.

To assess the differences in both the primary and secondary outcome variables based on preidentified subgroups, the following subgroups were generated: gender (divided into male, female, and transgender and gender-diverse people), age group (younger participants aged 16-20 years and older participants aged 21-25 years), and time spent on social media (moderate social media users who spent <5 hours on social media per day and high social media users who spent more than 5 hours on social media per day).

Perceived safety, conceptualized as adherence to the #chatsafe guidelines, was calculated using items from the Perceived Safety Questionnaire at T2 and reported as frequencies and percentages, with Fisher exact test values reported where comparisons between T1 and T2 have been made. Evaluations of the #chatsafe intervention content at T2 were reported as frequencies and percentages.

Statistical analyses were conducted using StataIC 15 (StataCorp LLC) [45].

### Ethics Approval and Safety

This study was approved by the University of Melbourne Human Research and Ethics Committee (ID: 1954623). In addition, several measures were taken to ensure participant safety. This included the development of an independent Safety Monitoring Committee to oversee study conduct, daily monitoring of all the #chatsafe social media accounts for any messages or comments that indicated distress, and monitoring of the weekly survey responses. Any distress reported by participants through contact with the study team or via responses to the weekly surveys was to be followed up within 24 hours. The participants were reminded that they were free to withdraw at any point and were also given the option of *snoozing* the weekly content, and this allowed them to take a 1-week break from the intervention. All correspondence to the participants included contact details of age-appropriate support services, such as eheadspace and Kids Helpline.

Finally, adverse events (AEs) and serious adverse events (SAEs) were monitored. In accordance with the organization’s policies, AEs were defined as any untoward or adverse effect related or unrelated to the study (eg, comments that expressed suicidal ideation). SAEs were defined as an event that resulted in death or as immediately life threatening or required hospitalization [46].

## Results

### Demographic Details

**Figure 3:**
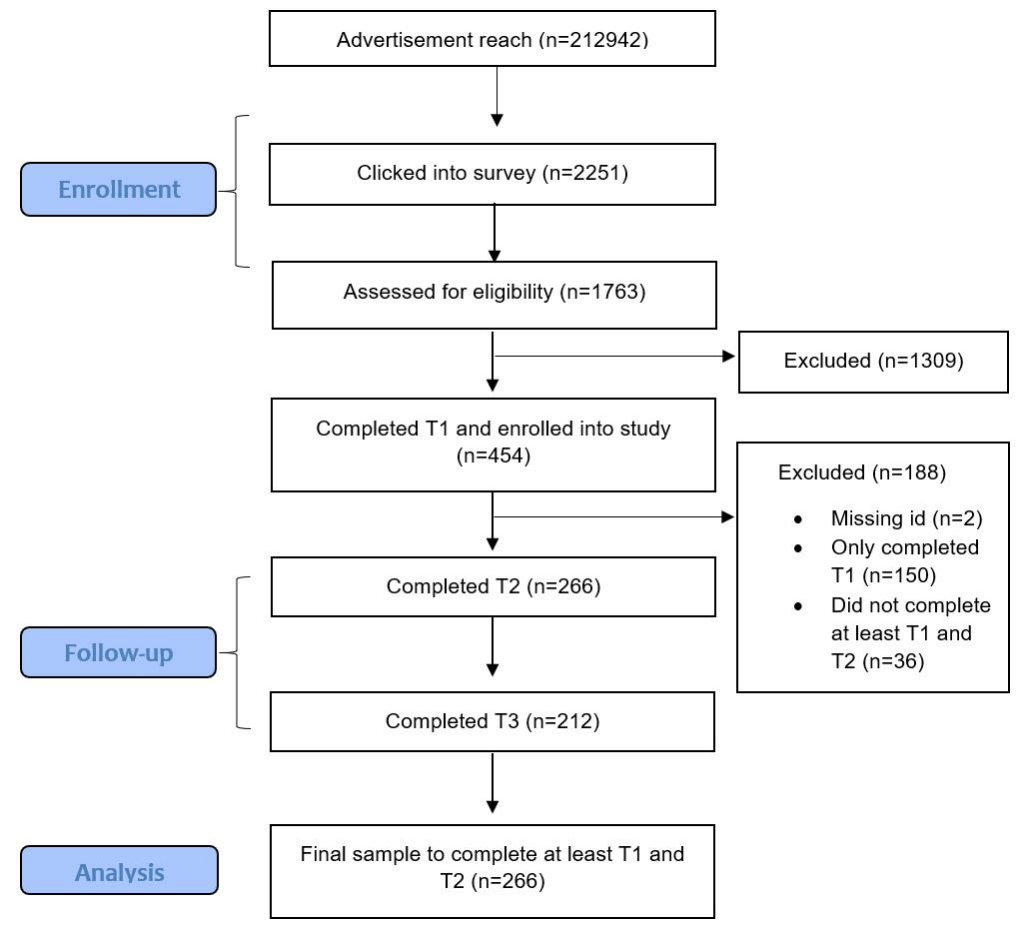
Participant flow diagram from enrollment to follow-up and data analysis.

As shown in Figure 3, a total of 1763 young people responded to the study advertisement and commenced eligibility screening; 454 young people were eligible and completed the T1 survey. Only participants who commenced the intervention and completed at least T1 and T2 were included in the analysis. This resulted in a final sample size of 266 and a retention rate of 58.59% across the study period.

The participant demographics are presented in Table 2. The participants were young adults aged between 16 and 25 years, with a median age of 18.9 years. Most of them (206/266, 77.4%) identified as cisgender female. More than half (145/266, 54.5%) of the sample identified as nonheterosexual, and the majority (213/266, 80.1%) were currently studying. Participants who did not complete the study and whose data were not retained in the final analysis did not significantly differ by age (*P*=.62), gender (*P*=.90), sexual orientation (*P*=.12), language (*P*=.55), Aboriginal and/or Torres Strait Islander descent (*P*=.95), student status (*P*=.64), relationship to someone who has attempted or died by suicide (*P*=.85), or social media use (*P*=.19)

**Table 2:** Demographic and baseline characteristics of participants who completed T1 and T2 (N=266).

Baseline characteristics		Values
Age (years), mean (SD)		18.9 (2.6)
**Gender, n (%)**		
	Male	32 (12)
	Female	206 (77.4)
	Transgender and gender-diverse participants	28 (10.5)
**Sexual orientation, n (%)**		
	Heterosexual (straight)	121 (45.5)
	Lesbian or gay	14 (5.3)
	Bisexual	74 (27.8)
	Other	57 (21.4)
**Language, n (%)**		
	English	240 (90.2)
	Other	26 (9.8)
**Aboriginal and/or Torres Strait Islander, n (%)**		
	Aboriginal	5 (1.9)
	Neither aboriginal nor Torres Strait Islander	261 (98.1)
**Currently studying, n (%)**		
	Yes	213 (80.1)
	No	53 (19.9)
**Relationship to someone who has attempted or died by suicide, n (%)**		
	Know in real life	234 (88)
	Know via the internet	32 (12)
**Social media use (hours), n (%)**		
	<1	4 (1.5)
	1-2	42 (15.8)
	2-3	80 (30.1)
	3-4	74 (27.8)
	≥5	66 (24.8)

The eligibility criteria meant that all participants had been exposed to a suicide or suicide attempt in the past 2 years. Most participants knew the person who had died by suicide or made a suicide attempt in their offline lives (234/266, 88%) as opposed to only knowing the person online.

### Social Media Use

Social media use among the participants was high. More than half (154/266, 57.9%) of the participants reported that they spent 2 to 4 hours per day on social media, and almost one-fourth (66/266, 24.8%) reported spending >5 hours per day on social media. The most commonly used platform was Instagram, followed by Snapchat, YouTube, Facebook, and Twitter. Tumblr was the least-used platform.

Exposure to suicide-related content on social media was common (Table 3).

**Table 3:** Types of suicide-related content seen by young people in the previous 4 weeks at each time point (N=266 at T1 and T2, N=212 at T3).

	T1, n (%)	T2, n (%)	T3, n (%)
Graphic descriptions of suicide	78 (29.3)	47 (17.7)	32 (15.1)
Graphic images of suicide	60 (22.6)	34 (12.8)	13 (6.1)
Means or methods of suicide	84 (31.6)	66 (24.8)	41 (19.3)
Plans of suicide	67 (25.2)	50 (18.8)	30 (14.2)
Statements that encourage people to take their own life	63 (23.7)	46 (17.3)	34 (16.0)
Statements that appear to deliberately seek to trigger difficult or distressing emotions in other people	108 (40.6)	74 (27.8)	60 (28.3)
Statements that include suicide pacts or suicide partners	27 (10.2)	17 (6.4)	16 (7.6)
Statements that place blame or make others feel responsible for another person’s safety	88 (33.1)	54 (20.3)	41 (19.3)
Statements that provide vulnerable people information about how to end their life	44 (16.5)	29 (10.9)	23 (10.9)
Suicide notes or goodbye notes	68 (25.6)	45 (16.9)	28 (13.2)
None	75 (28.2)	117 (44.0)	94 (44.3)

### Primary Outcome: Willingness to Intervene Against Suicide From T1 to T2

Table 4 presents the results of the logistic regression analysis that examined predictors of improvement and deterioration, relative to no change, in both subscales of the Willingness to Intervene Against Suicide measure from T1 to T2.

Most (154/266, 57.9%) participants showed substantial improvement in perceived behavioral control, almost one-fifth (50/266, 18.8%) showed deterioration, and almost one-quarter (62/266, 23.3%) showed no change. Baseline perceived behavioral control was associated with significant improvement from T1 to T2, whereby higher baseline scores reduced the likelihood of significant improvement (odds ratio [OR] 0.92, 95% CI 0.89-0.96; *P*<.001). No other predictor variables were associated with improvement in perceived behavioral control, and no predictor variables were associated with deterioration from T1 to T2.

Many (114/266, 42.9%) participants demonstrated improvement in intent to intervene, compared with 29.7% (79/266) of participants with no change in scores and 27.4% (73/266) who demonstrated deterioration. Of the potential predictors of improvement, only baseline intent to intervene scores were found to be significant, with higher baseline scores associated with a decrease in the likelihood of improvement (OR 0.90, 95% CI 0.87-0.95; *P*<.001). No other variables were associated with improvement in intent to intervene, and no variables were associated with deterioration from T1 to T2.

**Table 4:** Predictors of improvement and deterioration in the Willingness to Intervene Against Suicide (WIAS)-Perceived Behavioral Control (PBC) and Willingness to Intervene Against Suicide-Intent to Intervene T1 to T2.

Characteristics			Improvement^a^										Deterioration^a^							
			WIAS-PBC^b^					WIAS-Int^c^					WIAS-PBC			WIAS-Int				
			OR^d^ (95% CI)		*P* value		OR (95% CI)			*P* value		OR (95% CI)			*P* value			OR (95% CI)		*P* value
**Age (years)**																				
	<21	—^e^		—		—			—		—			—			—		—	
	≥21	0.99 (0.52-1.88)		.99		1.48 (0.78-2.80)			.23		0.64 (0.27-1.51)			.31			1.04 (0.50-2.15)		.92	
**Gender**																				
	Male	—		—		—			—		—			—			—		—	
	Female	2.25 (0.88-5.71)		.09		1.22 (0.47-3.17)			.69		1.10 (0.41-2.96)			.84			0.56 (0.22-1.42)		.23	
	Transgender and gender-diverse participants	1.63 (0.51-5.18)		.41		2.88 (0.75-11.10)			.12		0.41 (0.08-1.97)			.26			0.97 (0.23-4.04)		.97	
**Sexual orientation**																				
	Heterosexual or straight	—		—		—			—		—			—			—		—	
	Lesbian or gay	4.21 (0.52-34.21)		.18		1.53 (0.42-5.60)			.52		2.80 (0.24-33.04)			.41			1.02 (0.21-4.88)		.98	
	Bisexual	0.87 (0.43-1.74)		.69		1.61 (0.80-3.23)			.18		0.88 (0.35-2.22)			.79			1.43 (0.65-3.11)		.37	
	Other	0.74 (0.34-1.61)		.45		1.50 (0.69-3.27)			.31		1.60 (0.64-4.01)			.32			1.84 (0.80-4.22)		.15	
**Social media use (hours)**																				
	<5	—		—		—			—		—			—			—		—	
	≥5	1.03 (0.52-2.04)		.94		1.13 (0.58-2.19)			.72		1.10 (0.47-2.60)			.83			0.96 (0.45-2.03)		.91	
Baseline WIAS-PBC			*0.93 (0.91-0.96)* ^f^		*<.001*		—			—		1.02 (0.98-1.06)			.33			—		—
Baseline WIAS-Int			—		—		*0.93 (0.90-0.96)*			*<.001*		—			—			1.02 (0.99-1.06)		.25

^a^For both outcomes (improvement and deterioration), the comparator group consisted of participants who did not show a change in score over this period.

^b^WIAS-PBC: Willingness to Intervene Against Suicide–Perceived Behavioral Control.

^c^WIAS-Int: Willingness to Intervene Against Suicide–Intent to Intervene.

^d^OR: odds ratio.

^e^Row represents the reference group for the corresponding variable.

^f^Italicized values indicate significance.

### Secondary Outcomes

#### Willingness to Intervene From T1 to T3

Secondary analyses examining change in perceived behavioral control from T1 to T3 similarly found substantial improvement in most participants (139/212 65.57%); fewer than one-fifth demonstrated no change (35/212, 16.51%) or deterioration (38/212, 17.92%). Table S3 in Multimedia Appendix 1 shows the predictors of improvement in the perceived behavioral control subscale of the Willingness to Intervene Against Suicide measure.

A secondary analysis of the change from T1 to T3 indicated that half (104/212, 49.06%) of the sample were more likely to intervene, whereas approximately one-fourth demonstrated either no change (58/212, 27.36%) or deterioration (50/212, 23.58%). The predictors are presented in Table S3 in Multimedia Appendix 1.

#### Internet Self-efficacy

Approximately one-third of the participants demonstrated improved reactive self-efficacy (85/266, 32.2%), differentiation self-efficacy (79/266, 29.7%), and organizational self-efficacy (81/266, 30.45%), and approximately one-fifth demonstrated improvement in communication self-efficacy (55/266, 20.75%) and search self-efficacy (51/266, 19.25%). Most participants demonstrated no change in subdomains of the Internet Self-Efficacy scale. The predictors of improvement and deterioration are listed in Table S4 in Multimedia Appendix 1. Higher baseline scores in each of the subdomains were associated with a reduced likelihood of improvement for the corresponding subdomain, whereas higher baseline scores in the differentiation and search subdomains were associated with deterioration in the differentiation and search domains, respectively (Table S5 in Multimedia Appendix 1). Being aged ≥21 years was also associated with a reduced likelihood of deterioration by 53% (OR 0.43, 95% CI 0.20-0.93; *P*=.03) in the reactive subdomain.

##### Confidence and Safety (Adherence to the #chatsafe Guidelines) When Communicating Online About Suicide

At each time point, the participants were asked about their online experiences and behaviors in the preceding 4 weeks. Almost two-thirds of the sample reported that they had liked, shared, or created suicide-related content at T1 (173/266, 65.04%) and at T2 (179/266, 67.29%). Of these participants, the proportion that indicated that they monitored their posts for unsafe content increased from T1 (113/173, 65.32%) to T2 (149/179, 83.2%). Many participants reported not seeing unsafe content on their posts at both time points (T1: 46/113, 40.71% and T2: 67/149, 44.08%).

Only those who reported seeing unsafe content were asked how they dealt with that content. Participants most commonly reached out to the person who posted across both time points, although the proportion decreased from T1 to T2 (T1: 38/113, 33.63%; T2: 38/152, 25%). Participants also reported that they deleted (T1: 32/113, 28.32%; T2: 42/152, 27.63%) or hid the post (T1: 26/113, 23.01%; T2: 25/152, 16.45%). Some signposted helplines, although this was the least common response at both time points (T1: 17/113, 15.04%; T2: 18/152, 11.84%).

Among participants who encountered online content involving suicidal behavior that they found distressing, participants most commonly reported hiding certain posts on their feed (T1: 98/196, 50.00%; T2: 61/128, 47.66%) or taking a break from social media (T1: 77/196, 39.29%; T2: 60128, 46.88%), while approximately one-third of participants endorsed speaking to someone about how they were feeling at the time (T1: 65/196, 33.16%; T2: 44/128, 34.38%) or unfollowing the content from social media altogether (T1: 70/196, 35.71%; T2: 43/128, 33.59%)

Most participants reported seeing a post online that made them think the person was at risk of suicide, although rarely (T1: 221/266, 83.08%; T2: 195/266, 73.31%). Of these, more than half of the participants reported responding directly to the person (T1: 128/221, 57.92%; T2: 107/195, 54.87%). Many participants also endorsed informing a trusted friend or adult (T1: 44/221, 19.91%; T2: 47/195, 24.10%) or contacting the relevant platform safety center (T1: 39/221, 17.65%; T2: 40/195, 20.51%), and a minority reported seeking professional advice (T1: 12/221, 5.43%; T2: 22/195, 11.28%). At each time point, most participants indicated that they thought about whether they felt able to respond to the individual before deciding whether to respond (T1: 147/221, 66.52%; T2: 145/195, 74.36%).

##### Acceptability of the #chatsafe Intervention

###### Weekly Acceptability of Intervention Content

Overall, participants responded positively to the intervention content sent each week, and at no point was the intervention content deemed unsafe. Participants responded most positively to content from week 6, “How to check in on a friend who has been affected by suicide,” and responded least positively to content from week 5, “self-care.” Acceptability did not vary by gender, age group, or level of social media use (Table 5).

**Table 5:** Weekly acceptability of #chatsafe intervention content.

Week	Q1^a^		Q2^b^		Q3^c^		Total^d^
	Positive^e^, n (%)	Negative^f^, n (%)	Positive, n (%)	Negative, n (%)	Positive, n (%)	Negative, n (%)	
1	201 (90.95)	8 (3.62)	152 (68.77)	37 (16.74)	158 (71.49)	19 (8.59)	221
2	137 (95.81)	3 (2.1)	105 (73.43)	24 (16.79)	121 (84.61)	3 (2.10)	143
3	107 (83.59)	9 (7.03)	82 (64.07)	29 (22.65)	108 (84.38)	4 (3.12)	128
4	113 (87.60)	8 (6.21)	96 (74.42)	20 (15.51)	106 (82.17)	11 (8.53)	129
5	86 (69.92)	*15 (12.19)^g^*	69 (54.09)	*40 (32.53)*	90 (73.17)	*14 (11.39)*	123
6	*119 (96.75)*	2 (1.62)	*100 (81.30)*	14 (11.38)	*110 (89.43)*	4 (3.25)	123

^a^What did you think about the campaign content this week?

^b^Would you share this week’s campaign content with your contacts on social media?

^c^How did the campaign content you received today make you feel?

^d^Total number of responses received in that week.

^e^Positive sums were calculated by combining responses to ratings of 4 or 5 on a weekly emoji scale.

^f^Negative sums were calculated by combining responses to ratings of 1 or 2 on a weekly emoji scale [20].

^g^Italicized values indicate highest and lowest evaluations.

###### Postintervention Acceptability

Almost half (132/266, 49.62%) of the participants reported finding the #chatsafe content to be helpful. Almost half (126/266, 47.37%) of the participants reported that the intervention material made them feel more confident when talking about suicide online. Most participants reported that the #chatsafe content posed no risk to themselves (254/266, 95.49%), and they did not feel that it would be a risk to others (224/266, 84.21%). More than one-third (106/266, 39.85%) of participants believed that the #chatsafe content would help prevent further suicide or suicide attempts in others following an index suicide in the community.

##### Safety of the #chatsafe Intervention

No AEs or SAEs were observed during the study period. A total of 32 people were lost to follow up throughout the study period (ie, they changed their social media handle, deactivated their social media account, or unfollowed the #chatsafe profile and therefore could not be contacted). Across the 6-week intervention, 3 participants requested to *snooze* the content for a period of 1 week. None of the participants expressed distress or requested that a member of the study team contact them at any stage of the study.

## Discussion

### Principal Findings

The aim of this study was to explore the role social media can play in supporting young people who have been exposed to a suicide or a suicide attempt by testing the impact of the #chatsafe intervention. The findings from this study not only support the safety, acceptability, and impact of the #chatsafe intervention but also point to an increase in participants’ willingness to intervene against suicide online. The findings suggest that the #chatsafe intervention may have increased some young people’s internet self-efficacy as well as their confidence and safety when communicating online about suicide. Although most participants reported improvements in the primary and secondary outcome variables, they appeared to be quite proficient in safe communication practices at baseline, with high scores on perceived internet self-efficacy and a strong endorsement of items from the #chatsafe guidelines [26]. Although this limited the rate at which improvement on these outcomes could be measured, the findings from this study support the utility of using social media to reach young people with suicide prevention information.

Young people are frequently exposed to suicide-related content online, and it is well documented that exposure can increase the risk of future suicide and suicide-related behavior [8,9]. Almost two-thirds of the participants in this study had liked, shared, or created suicide-related content on social media, and the majority had seen posts online which made them think someone was at risk of suicide. High rates of exposure to content such as information about methods of suicide, statements that participants felt were deliberately attempting to trigger difficult or distressing emotions, and statements that made others feel responsible for someone’s safety were also recorded. Approximately one-fourth of the sample had seen suicide notes, comments encouraging suicide, and graphic images of suicide. This is of concern, considering that harmful content, such as specific details about suicide, is thought to encourage imitative behavior [14,34,47]. These data speak to the amount and type of suicide-related content that young people are exposed to online and add further weight to the growing concerns about the potential impact of social media on youth mental health and suicide risk [9,16,47]. Taken together, these data highlight the importance of equipping young people with the skills to keep themselves and their peers safe when actively or passively engaging with suicide-related information on social media. They also support social media being an important context to consider when implementing an effective postvention response for young people [20,32,48].

### Implications

Findings from this study suggest that the #chatsafe intervention achieved its objective of educating young people about the importance of safe online communication about suicide and provides an example of how social media content could be incorporated into a postvention approach. The greatest increases were observed in participants’ perceived behavioral control to respond to suicide-related content online, suggesting that the #chatsafe intervention increased their belief in their ability to safely manage or intervene against suicide-related content. Equipping young people with the knowledge to keep themselves and others safe is the primary goal of #chatsafe and ensuring that young people feel able to share and respond to suicide-related content safely is the first step. However, despite most participants reporting a greater confidence in their ability to respond to suicide-related content after receiving the #chatsafe intervention, there was a lesser increase in young people’s intention to respond, and for a third of the sample, there was a decrease. In other words, possessing the confidence to communicate safely about suicide may not lead to actually engaging in a safe response or communication. This is not an uncommon finding in evaluations of mass media campaigns for suicide prevention, where raising awareness does not always translate to behavior change [49]. Alternatively, and perhaps more likely, the information provided by the #chatsafe intervention may have dissuaded young people from engaging in online conversations about suicide altogether, particularly if they were better able to assess the content that they come across as unsafe. The types of suicide-related content that participants reported seeing on social media suggest that they are mostly exposed to concerning content about this topic, and there is a chance that the information contained within the #chatsafe intervention empowered young people to disengage, block, or report that content rather than feeling the need to intervene.

The #chatsafe intervention provided general psychoeducation around suicide and digital literacy, and there was no heavy focus on encouraging young people to directly respond to suicide-related content online. A key message within the #chatsafe guidelines is for young people to check in with themselves and not feel the sole responsibility of engaging in conversations about suicide with someone that they are worried about. Despite often having the best intentions, some research suggests that young people who provide support about suicide or self-harm to others via social media report feeling worse themselves after that interaction [50]. However, the most preferred piece of content during this study included specific examples of how young people can approach a conversation about suicide, such as “it’s okay to feel that way” and “How do you feel about meeting for a coffee?” Although these are simple statements, guidance about what to say, or examples of words to use, likely address common fears about “saying the wrong thing” and may serve to protect those who would like to offer others support but feel ill-equipped to do so. This is a major gap in the current body of resources available to young people and one that future iterations of the #chatsafe intervention will attempt to address.

Most importantly, the #chatsafe intervention appeared to be safe and no adverse reactions were recorded. In addition, 97% of the participants reported that the content did not pose a risk to themselves, and 87% felt that it would not be a risk for others. That said, this study only retained approximately 60% of the participants throughout the intervention period, and although retaining this proportion of young people in repeated measures studies is not uncommon [51,52], the findings should be interpreted with caution. While none of the participants indicated distress upon withdrawal, it is possible that some participants found the content overwhelming or unhelpful, which may be reflected in the finding that 40% of the participants did not believe that the content would be helpful in preventing future suicide deaths. This is unsurprising, as suicide is complex and unlikely to be prevented by a single intervention.

These findings support the potential for a social media intervention to play an important role in a broader postvention strategy, with a focus on disseminating age-appropriate and helpful information to young people. It has been recommended that after a suicide, postvention strategies aimed at mitigating suicide clusters need to be multifaceted and include a range of different approaches, including the monitoring of social media [28,30,32,33]. After a suicide has occurred, a social media intervention, such as #chatsafe, may result in safer online communication about suicide and subsequently act as a protective factor for young people in that online community [53]. Indeed, the outcomes of this study have already had practical implications for postvention responses delivered in real time and via social media. Since this study was conducted, the #chatsafe intervention material has been disseminated across communities in Australia (Western Australia, Victoria, and New South Wales) and New Zealand following a youth suicide. So far, at the time of writing, these interventions have reached ≥800,000 young people, and it is hoped that the #chatsafe content has contributed toward safer communication and the sharing of helpful information within those communities.

### Strengths and Limitations

A key strength of this study was that it involved the delivery of a youth co-designed suicide prevention intervention shared within the environment in which young people are likely to encounter suicide-related information. Delivering interventions via social media makes them accessible, easily distributed, and relatively cost-effective [20]. It is also possible to reach large numbers of individuals in a short span of time. While young people at an elevated risk of suicide have historically been underrepresented in youth suicide prevention research [54], this study specifically recruited young people who had been impacted by a suicide or suicide attempt, a group known to be overrepresented in the suicide statistics. Furthermore, the study attracted a larger proportion of LGBTQIA+ (lesbian, gay, bisexual, transgender, queer, intersex, asexual) young people than the general population, another group who are disproportionately affected by suicide [55]. Despite the recruitment of young people within these groups, participants were predominantly cisgender females, and more work is required to understand the impact of the intervention on different groups of young people, particularly young males.

As this study was novel in its approach and the first of its kind, there are several learnings for future suicide prevention interventions delivered via social media. First, this was not a controlled study, and the changes observed cannot be directly attributed to the #chatsafe intervention. While this was a pilot study, a randomized controlled trial of the #chatsafe intervention commenced in November 2022 (Trial ID: ACTRN12622001397707). Second, this study did not collect information about the timing of suicide bereavement or exposure to a suicide attempt (other than it being within the past 2 years) nor did we collect information about the proximity to the suicide death or the subjective relationship with the deceased. This information is required to more thoroughly explore how the grieving process might impact the way in which #chatsafe content is perceived by young people. Third, although our questionnaire comprised measures and scales previously validated in other youth samples, they were not specifically designed to assess adherence to the #chatsafe guidelines and may not have adequately captured online behaviors and experiences relevant to the #chatsafe guidelines. This may account for the lack of predictor variables identified in our analyses. The ongoing randomized controlled trial using the #chatsafe intervention will use a new questionnaire that is tailored to measure adherence to the #chatsafe guidelines and more accurately address our research questions.

Previous work has identified that changes in willingness to intervene against suicide may be influenced by the type of exposure to suicide [56]. Participants in this study were eligible if they knew someone who had died by suicide and if they knew of a suicide attempt. Experiencing a suicide death versus knowing someone affected by suicide are qualitatively different experiences that are likely to impact the way one communicates about suicide and the way they are impacted by the communication of others [57]. Furthermore, the Circles of Vulnerability Model would argue that the degree of emotional impact felt by a suicide death is contingent upon 3 factors: geographical proximity, psychosocial proximity, and population at risk [11], yet little work has explored the role social media plays in determining proximity to suicide or in determining the closeness felt toward suicide-related content. Future research should seek to understand the differences in exposure and proximity (both online and offline) to develop and disseminate the most appropriate postvention material at the right time. Third, providing support to someone online is likely to be different from the offline context, and furthermore, recognizing and responding to risks may also be more challenging. It has previously been reported that perceptions of risk severity were a key factor influencing intent to intervene with a suicidal peer [58]. Observing others’ social media behavior is largely subjective, and this may make it a particularly challenging environment to offer support. Future research should explore the ways young people subjectively perceive distress or risk on social media so that interventions, such as the #chatsafe intervention, can best reflect the needs and wishes of young people.

Finally, this study found that young people are frequently exposed to harmful suicide-related content online. Although the guidance provided by the #chatsafe intervention aims to equip young people with the skills to communicate safely online about suicide, more information is needed to understand the impact of exposure (and at times, multiple exposures) on young people, particularly in relation to their own mental health. Further investigation of individual differences in the perception of risk and subsequent responses to suicide-related content will allow for more tailored intervention content for specific groups of young people in the future.

### Conclusions

The findings of this study suggest that it is safe and acceptable to deliver a social media–based suicide prevention intervention to young people who have been exposed to a suicide or suicide attempt. The #chatsafe intervention social media content was received positively, and after exposure to the intervention, many participants reported a greater willingness to intervene against suicide, as well as increases in their perceived internet self-efficacy, confidence, and safety when communicating on social media about suicide. This was the first study to exclusively test the acceptability, impact, and safety of a suicide prevention social media intervention with a sample of recently bereaved young people. This study has provided preliminary evidence that #chatsafe is a safe and potentially efficacious intervention that could form part of future postvention responses and, as such, may have the potential to help reduce the risk of imitation or contagion after a suicide has occurred.

## Appendix

Multimedia Appendix 1 Report on change thresholds and predictors.

## Abbreviations

AE: adverse event
LGBTQIA+: lesbian, gay, bisexual, transgender, queer, intersex, asexual
OR: odds ratio
SAE: serious adverse event
TIDieR: Template for Intervention Description and Replication
