# Supplementary material for: Acceptability and Potential Impact of the #chatsafe Suicide Postvention Response Among Young People Who Have Been Exposed to Suicide: Pilot Study
Source: JMIR Hum Factors. 2023 May 19;10:e44535. doi: 10.2196/44535 (PMC10238962; doi:10.2196/44535)
Supplement: Multimedia Appendix 1 [file humanfactors_v10i1e44535_app1.docx]

**Supplementary File**

Table S1. Change thresholds by measure, T1 to T2

| Measure | SD | Absolute threshold of change score | Interval of change scores for ‘significant improvement’ | | Interval of change scores for ‘no significant change’ | | Interval of change scores for ‘significant deterioration’ | |
| --- | --- | --- | --- | --- | --- | --- | --- | --- |
|  |  |  | Max | Min | Max | Min | Max | Min |
| WIAS-PBC | 12.6 | 3 | 80 | 4 | 3 | -3 | -80 | -4 |
| WIAS-INT | 11.3 | 3 | 88 | 4 | 3 | -3 | -88 | -4 |
| ISS-R | 7.0 | 3 | 24 | 4 | 3 | -3 | -24 | -4 |
| ISS-D | 4.8 | 2 | 16 | 3 | 2 | -2 | -3 | -16 |
| ISS-O | 3.1 | 1 | 12 | 2 | 1 | -1 | -2 | -12 |
| ISS-C | 2.4 | 1 | 8 | 2 | 1 | -1 | -2 | -8 |
| ISS-S | 1.7 | 1 | 8 | 2 | 1 | -1 | -2 | -8 |

Table S2. Change thresholds by measure, T1 to T3

| Measure | SD | Absolute threshold of change score | Interval of change scores for ‘significant improvement’ | | Interval of change scores for ‘no significant change’ | | Interval of change scores for ‘significant deterioration’ | |
| --- | --- | --- | --- | --- | --- | --- | --- | --- |
|  |  |  | Max | Min | Max | Min | Max | Min |
| WIAS-PBC | 12.2 | 3 | 80 | 4 | 3 | -3 | -80 | -4 |
| WIAS-INT | 11.1 | 3 | 88 | 4 | 3 | -3 | -88 | -4 |
| ISS-R | 6.7 | 3 | 24 | 4 | 3 | -3 | -24 | -4 |
| ISS-D | 4.4 | 2 | 16 | 3 | 2 | -2 | -3 | -16 |
| ISS-O | 2.9 | 1 | 12 | 2 | 1 | -1 | -2 | -12 |
| ISS-C | 2.5 | 1 | 8 | 2 | 1 | -1 | -2 | -8 |
| ISS-S | 1.7 | 1 | 8 | 2 | 1 | -1 | -2 | -8 |

Table S3. Predictors of improvement and deterioration in WIAS-PBC and WIAS-Int, T1 to T3

|  | Improvement |  |  |  |  |  | Deterioration |  |  |  |  |  |
| --- | --- | --- | --- | --- | --- | --- | --- | --- | --- | --- | --- | --- |
| Characteristic | WIAS-PBC |  |  | WIAS-Int |  |  | WIAS-PBC |  |  | WIAS-Int |  |  |
|  | OR | 95% CI | P-value | OR | 95% CI | P-value | OR |  | P-value | OR |  | P-value |
| Age group |  |  |  |  |  |  |  |  |  |  |  |  |
| <21 |  |  |  |  |  |  |  |  |  |  |  |  |
| 21+ | 0.91 | 0.41-2.03 | 0.823 | 1.06 | 0.51-2.19 | 0.883 | 0.41 | 0.13-1.26 | 0.120 | 1.23 | 0.53-2.86 | 0.632 |
| Gender |  |  |  |  |  |  |  |  |  |  |  |  |
| Male |  |  |  |  |  |  |  |  |  |  |  |  |
| Female | 2.26 | 0.71-7.20 | 0.167 | 1.39 | 0.46-4.20 | 0.563 | 0.60 | 0.18-2.03 | 0.412 | 0.88 | 0.29-2.65 | 0.822 |
| Trans and gender diverse | 1.60 | 0.37-6.96 | 0.531 | 4.69 | 0.72-30.58 | 0.106 | 0.22 | 0.03-1.59 | 0.135 | 3.43 | 0.52-22.80 | 0.202 |
| Sexual orientation |  |  |  |  |  |  |  |  |  |  |  |  |
| Heterosexual/straight |  |  |  |  |  |  |  |  |  |  |  |  |
| Lesbian or gay | - |  |  | 1.56 | 0.29-8.28 | 0.602 | - | - | - | 1.70 | 0.26-11.06 | 0.581 |
| Bisexual | 1.07 | 0.44-2.64 | 0.881 | 0.89 | 0.41-1.90 | 0.758 | 1.01 | 0.31-3.27 | 0.990 | 0.60 | 0.22-1.60 | 0.306 |
| Other | 0.70 | 0.28-1.79 | 0.461 | 0.76 | 0.32-1.77 | 0.527 | 1.64 | 0.55-4.91 | 0.379 | 1.30 | 0.51-3.31 | 0.576 |
| Social media usage |  |  |  |  |  |  |  |  |  |  |  |  |
| Less than 5 hours |  |  |  |  |  |  |  |  |  |  |  |  |
| 5 or more hours | 1.05 | 0.44-2.53 | 0.912 | 2.39 | 0.95-6.00 | 0.063 | 1.56 | 0.55-4.43 | 0.405 | 2.20 | 0.82-5.84 | 0.115 |
| Baseline WIAS-PBC | **0.94** | **0.91-0.98** | **0.001** |  |  |  | 1.00 | 0.96-1.05 | 0.876 |  |  |  |
| Baseline WIAS-Int |  |  |  | **0.93** | **0.89-0.96** | **<0.001** |  |  |  | 1.00 | 0.96-1.04 | 0.848 |

Table S4. Predictors of improvement and deterioration in domains of ISS, T1 to T3

|  | Improvement |  |  |  |  |  |  |  |  |  | Deterioration |  |  |  |  |  |  |  |  |  |
| --- | --- | --- | --- | --- | --- | --- | --- | --- | --- | --- | --- | --- | --- | --- | --- | --- | --- | --- | --- | --- |
| Characteristic | ISS-R |  | ISS-D |  | ISS-O |  | ISS-C |  | ISS-S |  | ISS-R |  | ISS-D |  | ISS-O |  | ISS-C |  | ISS-S |  |
|  | OR (95% CI) | P-value | OR (95% CI) | P-value | OR (95% CI) | P-value | OR (95% CI) | P-value | OR (95% CI) | P-value | OR (95% CI) | P-value | OR (95% CI) | P-value | OR (95% CI) | P-value | OR (95% CI) | P-value | OR (95% CI) | P-value |
| Age group |  |  |  |  |  |  |  |  |  |  |  |  |  |  |  |  |  |  |  |  |
| <21 |  |  |  |  |  |  |  |  |  |  |  |  |  |  |  |  |  |  |  |  |
| 21+ | 1.21 (0.63-2.32) | 0.575 | 0.57 (0.28-1.19) | 0.136 | 0.55 (0.27-1.12) | 0.101 | 0.86 (0.42-1.78) | 0.683 | 0.68 (0.27-1.71) | 0.415 | 0.58 (0.22-1.56) | 0.281 | 0.68 (0.30-1.55) | 0.359 | 0.50 (0.22-1.13) | 0.097 | 0.60 (0.25-1.44) | 0.253 | 0.71 (0.32-1.57) | 0.398 |
| Gender |  |  |  |  |  |  |  |  |  |  |  |  |  |  |  |  |  |  |  |  |
| Male |  |  |  |  |  |  |  |  |  |  |  |  |  |  |  |  |  |  |  |  |
| Female | 0.74 (0.29-1.86) | 0.518 | 0.44 (0.17-1.16) | 0.097 | 1.82 (0.60-5.45) | 0.287 | 0.76 (0.28-2.03) | 0.581 | 0.67 (0.22-2.02) | 0.478 | 1.21 (0.31-4.65) | 0.784 | 0.68 (0.21-2.16) | 0.508 | 0.79 (0.28-2.18) | 0.645 | 1.62 (0.44-6.00) | 0.469 | 1.88 (0.52-6.82) | 0.337 |
| Trans and gender diverse | 0.73 (0.21-2.54) | 0.624 | 0.60 (0.17-2.14) | 0.430 | 1.44 (0.34-6.17) | 0.623 | 1.64 (0.46-5.80) | 0.445 | 0.80 (0.18-3.54) | 0.769 | 0.92 (0.12-5.53) | 0.924 | 0.45 (0.08-2.40) | 0.349 | 1.20 (0.31-4.59) | 0.790 | 1.27 (0.21-7.58) | 0.791 | 1.00 (0.17-5.72) | 1.000 |
| Sexual orientation |  |  |  |  |  |  |  |  |  |  |  |  |  |  |  |  |  |  |  |  |
| Heterosexual/straight |  |  |  |  |  |  |  |  |  |  |  |  |  |  |  |  |  |  |  |  |
| Lesbian or gay | 0.52 (0.10-2.74) | 0.441 | 1.10 (0.25-4.76) | 0.898 | 0.68 (0.16-2.84) | 0.596 | 0.40 (0.05-3.47) | 0.403 | 1.42 (0.26-7.76) | 0.684 | 1.56 (0.35-6.97) | 0.559 | 1.02 (0.19-5.50) | 0.983 | 0.29 (0.03-2.46) | 0.25 | 2.24 (0.56-8.85) | 0.252 | 1.68 (0.38-7.38) | 0.489 |
| Bisexual | 1.33 (0.65-2.71) | 0.431 | 1.57 (0.75-3.31) | 0.233 | 1.33 (0.63-2.80) | 0.449 | 1.07 (0.49-2.37) | 0.862 | 0.90 (0.35-2.32) | 0.824 | 0.58 (0.19-1.75) | 0.333 | 0.76 (0.29-2.04) | 0.592 | 0.72 (0.29-1.79) | 0.480 | 1.08 (0.44-2.65) | 0.864 | 0.80 (0.33-1.94) | 0.618 |
| Other | 1.14 (0.52-2.48) | 0.749 | 1.43 (0.62-3.32) | 0.406 | 1.23 (0.53-2.85) | 0.624 | 1.19 (0.52-2.69) | 0.681 | 0.88 (0.31-2.51) | 0.815 | 1.28 (0.49-3.33) | 0.615 | 2.14 (0.90-5.08) | 0.085 | 1.67 (0.71-3.89) | 0.238 | 1.03 (0.40-2.69) | 0.949 | 1.39 (0.60-3.25) | 0.441 |
| Social media usage |  |  |  |  |  |  |  |  |  |  |  |  |  |  |  |  |  |  |  |  |
| Less than 5 hours |  |  |  |  |  |  |  |  |  |  |  |  |  |  |  |  |  |  |  |  |
| 5 or more hours | 1.55 (0.79-3.04) | 0.199 | 1.93 (0.95-3.92) | 0.070 | 1.35 (0.67-2.73) | 0.402 | 1.85 (0.89-3.82) | 0.099 | 1.09 (0.45-2.68) | 0.841 | 0.75 (0.28-2.03) | 0.571 | 1.45 (0.63-3.34) | 0.383 | 0.84 (0.36-1.93) | 0.674 | 1.49 (0.65-3.41) | 0.341 | 1.22 (0.56-2.64) | 0.614 |
| Baseline ISS-R | **0.84 (0.79-0.89)** | **<0.001** |  |  |  |  |  |  |  |  | 1.05 (0.98-1.12) | 0.212 |  |  |  |  |  |  |  |  |
| Baseline ISS-D |  |  | **0.76 (0.68-0.84)** | **<0.001** |  |  |  |  |  |  |  |  | **1.03 (0.94-1.13)** | **0.545** |  |  |  |  |  |  |
| Baseline ISS-O |  |  |  |  | **0.51 (0.41-0.62)** | **<0.001** |  |  |  |  |  |  |  |  | 0.96 (0.82-1.13) | 0.622 |  |  |  |  |
| Baseline ISS-C |  |  |  |  |  |  | **0.51 (0.41-0.63)** | **<0.001** |  |  |  |  |  |  |  |  | **1.28 (1.00-1.63)** | **0.047** |  |  |
| Baseline ISS-S |  |  |  |  |  |  |  |  | **0.46 (0.34-0.61)** | **<0.001** |  |  |  |  |  |  |  |  | 1.05 (0.98-1.12) | 0.212 |

Table S5. Predictors of improvement and deterioration in subdomains of ISS, T1 to T3

|  | Improvement | | | | | | | | | | Deterioration | | | | | | | | |  |
| --- | --- | --- | --- | --- | --- | --- | --- | --- | --- | --- | --- | --- | --- | --- | --- | --- | --- | --- | --- | --- |
| **Characteristic** | ISS-R |  | ISS-D |  | ISS-O |  | ISS-C |  | ISS-S |  | ISS-R |  | ISS-D |  | ISS-O |  | ISS-C |  | ISS-S |  |
|  | OR (95% CI) | P-value | OR (95% CI) | P-value | OR (95% CI) | P-value | OR (95% CI) | P-value | OR (95% CI) | P-value | OR (95% CI) | P-value | OR (95% CI) | P-value | OR (95% CI) | P-value | OR (95% CI) | P-value | OR (95% CI) | P-value |
| **Age group** |  |  |  |  |  |  |  |  |  |  |  |  |  |  |  |  |  |  |  |  |
| <21 |  |  |  |  |  |  |  |  |  |  |  |  |  |  |  |  |  |  |  |  |
| 21+ | 0.63 (0.35-1.17) | 0.144 | 0.69 (0.37-1.28) | 0.237 | 1.19 *0.65-2.19) | 0.578 | 1.15 (0.59-2.24) | 0.684 | 0.99 (0.50-1.96) | 0.969 | **0.43 (0.20-0.93)** | **0.033** | 0.66 (0.33-1.33) | 0.246 | 0.87 (0.44-1.74) | 0.704 | 1.11 (0.56-2.20) | 0.763 | 0.85 (0.42-1.75) | 0.668 |
| **Gender** |  |  |  |  |  |  |  |  |  |  |  |  |  |  |  |  |  |  |  |  |
| Male |  |  |  |  |  |  |  |  |  |  |  |  |  |  |  |  |  |  |  |  |
| Female | 0.71 (0.31-1.67) | 0.436 | 0.99 (0.42-2.32) | 0.985 | 0.70 (0.29-1.69) | 0.427 | 0.68 (0.27-1.68) | 0.401 | 0.69 (0.28-1.71) | 0.424 | 0.82 (0.31-2.21) | 0.699 | 1.17 (0.43-3.20) | 0.755 | 0.70 (0.28-1.78) | 0.455 | 1.10 (0.41-2.94) | 0.853 | 1.23 (0.43-3.49) | 0.702 |
| Trans and gender diverse | 0.70 (0.22-2.19) | 0.537 | 0.80 (0.24-2.66) | 0.716 | 0.61 (0.19-1.95) | 0.408 | 1.41 (0.45-4.43) | 0.561 | 0.75 (0.22-2.58) | 0.648 | 0.66 (0.17-2.62) | 0.558 | 1.33 (0.36-4.92) | 0.666 | 0.25 (0.06-1.13) | 0.071 | 0.38 (0.07-2.13) | 0.268 | 0.60 (0.13-2.87) | 0.523 |
| **Sexual orientation** |  |  |  |  |  |  |  |  |  |  |  |  |  |  |  |  |  |  |  |  |
| Heterosexual/straight |  |  |  |  |  |  |  |  |  |  |  |  |  |  |  |  |  |  |  |  |
| Lesbian or gay | 0.67 (0.19-2.39) | 0.539 | 1.36 (0.40-4.63) | 0.621 | 2.10 (0.56-7.81) | 0.268 | 0.71 (0.14-3.57) | 0.676 | 0.65 (0.13-3.21) | 0.596 | 0.41 (0.08-2.06) | 0.280 | 0.62 (0.12-3.19) | 0.570 | 1.80 (0.45-7.21) | 0.407 | 1.21 (0.34-4.36) | 0.766 | 1.11 (0.28-4.44) | 0.887 |
| Bisexual | 1.04 (0.54-1.99) | 0.917 | 1.22 (0.62-2.39) | 0.568 | 1.90 (0.97-3.71) | 0.061 | 0.96 (0.46-2.01) | 0.923 | 0.89 (0.43-1.86) | 0.765 | 0.56 (0.26-1.25) | 0.157 | 0.91 (0.43-1.92) | 0.800 | 1.09 (0.51-2.33) | 0.827 | 0.57 (0.26-1.25) | 0.161 | 0.68 (0.30-1.55) | 0.358 |
| Other | 0.72 (0.34-1.50) | 0.374 | 1.39 (0.67-2.89) | 0.373 | 1.58 (0.74-3.33) | 0.235 | 1.08 (0.49-2.39) | 0.843 | 0.77 (0.33-1.83) | 0.559 | 0.55 (0.24-1.27) | 0.163 | 1.01 (0.44-2.28) | 0.990 | 1.41 (0.64-3.08) | 0.394 | 0.71 (0.31-1.64) | 0.428 | 1.37 (0.62-2.99) | 0.435 |
| **Social media usage** |  |  |  |  |  |  |  |  |  |  |  |  |  |  |  |  |  |  |  |  |
| Less than 5 hours |  |  |  |  |  |  |  |  |  |  |  |  |  |  |  |  |  |  |  |  |
| 5 or more hours | 0.80 (0.43-1.50) | 0.491 | 1.21 (0.63-2.30) | 0.568 | 0.78 (0.40-1.51) | 0.458 | 1.12 90.57-2.22) | 0.730 | 0.87 (0.42-1.82) | 0.717 | 0.38 (0.16-0.89) | 0.027 | 1.19 (0.58-2.43) | 0.634 | 0.87 (0.43-1.76) | 0.700 | 0.49 (0.21-1.12) | 0.089 | 0.82 (0.39-1.75) | 0.610 |
| Baseline ISS-R | **0.82 (0.78-0.87** | **<0.001** |  |  |  |  |  |  |  |  | 1.03 (0.98-1.10) | 0.192 |  |  |  |  |  |  |  |  |
| Baseline ISS-D |  |  | **0.76 (0.69-0.82)** | **<0.001** |  |  |  |  |  |  |  |  | **1.15 (1.04-1.26)** | **0.007** |  |  |  |  |  |  |
| Baseline ISS-O |  |  |  |  | **0.58 (0.49-0.67)** | **<0.001** |  |  |  |  |  |  |  |  | 1.05 (0.92-1.21) | 0.454 |  |  |  |  |
| Baseline ISS-C |  |  |  |  |  |  | **0.55 (0.46-0.66)** | **<0.001** |  |  |  |  |  |  |  |  | 1.13 (0.95-1.34) | 0.170 |  |  |
| Baseline ISS-S |  |  |  |  |  |  |  |  | **0.44 (0.34-0.57)** | **<0.001** |  |  |  |  |  |  |  |  | **1.51 (1.09-2.10)** | **0.012** |
